# User journeys in cross-European secondary use of health data: insights ahead of the European Health Data Space

**DOI:** 10.1093/eurpub/ckaf096

**Published:** 2025-09-10

**Authors:** Rachel B Forster, Eva Garcia Alvarez, Adrian G Zucco, Enrique Bernal-Delgado, Gayo Diallo, Francisco Estupiñán-Romero, Andrea Ganna, Madeleine Gorman-Asal, Christina Hilmarsen, Petr Holub, Klaus Hoeyer, Arti Rawat, Naja H Rod, Anna-Leena Vuorinen, Tibor V Varga

**Affiliations:** Department of Health Registry Research and Development, Norwegian Institute of Public Health, Bergen, Norway; Biobanking and Biomolecular Resources Research Infrastructure (BBMRI-ERIC), IT department, Graz, Austria; Copenhagen Health Complexity Center, Department of Public Health, University of Copenhagen, Copenhagen, Denmark; Data Science for Health Services and Policy Research Group, Aragon Health Sciences Institute (IACS), Zaragoza, Spain; Team AHead, Bordeaux Population Health INSERM-U1219, Univ. Bordeaux, Bordeaux, France; Data Science for Health Services and Policy Research Group, Aragon Health Sciences Institute (IACS), Zaragoza, Spain; Institute for Molecular Medicine Finland (FIMM), HiLIFE, University of Helsinki, Helsinki, Finland; Broad Institute of MIT and Harvard, Cambridge, MA, United States; Analytic and Translational Genetics Unit, Massachusetts General Hospital, Boston, MA, United States; Team AHead, Bordeaux Population Health INSERM-U1219, Univ. Bordeaux, Bordeaux, France; New Brunswick Institute for Research, Data and Training, University of New Brunswick, Fredericton, NB, Canada; Norwegian Institute of Public Health, Health Data Service, Tynset, Norway; Biobanking and Biomolecular Resources Research Infrastructure (BBMRI-ERIC), IT department, Graz, Austria; Institute of Computer Science, Masaryk University, Brno, Czech Republic; Section for Health Services Research, Department of Public Health, University of Copenhagen, Copenhagen, Denmark; Team AHead, Bordeaux Population Health INSERM-U1219, Univ. Bordeaux, Bordeaux, France; Copenhagen Health Complexity Center, Department of Public Health, University of Copenhagen, Copenhagen, Denmark; Institute for Molecular Medicine Finland (FIMM), HiLIFE, University of Helsinki, Helsinki, Finland; Unit of Health Sciences, Faculty of Social Sciences, Tampere University, Tampere, Finland; Copenhagen Health Complexity Center, Department of Public Health, University of Copenhagen, Copenhagen, Denmark

## Abstract

The European Health Data Space (EHDS) regulation aims to facilitate cross-border sharing of health data across Europe. However, practical challenges related to data access, interoperability, quality, and interpretive competence remain, particularly when working with health systems across countries. This study aimed to evaluate and report the user journey of researchers accessing and utilizing health data across four European countries for secondary research purposes prior to implementation of EHDS. We conducted a narrative reflection of individual and collective experiences on key aspects of the user journey—discovery, access, use, and finalization. Data were gathered from various structured and unstructured sources, including an online log, prospective questionnaires, regular meetings, and interviews. Researchers faced challenges at different steps of the user journey, which included lack of data quality in national metadata catalogues (discovery stage). Differences in national regulations led to inconsistent timelines for gaining access to data (access stage), with approval times ranging from a few months to over a year. At the use stage, researchers experienced challenges in harmonizing health data due to variations in coding practices and data quality. Issues related to computational capacity caused further delays. Substantial challenges must be addressed for EHDS to succeed. Establishing knowledge hubs, fostering collaborations, and streamlining access processes are essential. Close collaboration with experts will likely be essential for an effective user journey. This analysis underscores the importance of collaboration, analytical reproducibility, and clear documentation to ensure the success and timely delivery of cross-border projects.

## Introduction

In 2024, the European Parliament and the Council reached a political agreement on the European Health Data Space (EHDS) regulation proposal, which was adopted by the European Council in January 2025 (latest version: Brussels, 8 January 2025; PE-CONS 76/24; 2022/0140(COD)) [1]. The EHDS is a governing framework that aims to harmonize the landscape of health data utilization in the European Union (EU) through a vision of a secure and collaborative ecosystem and a cross-border infrastructure where health data across European Union Member States can be shared for primary and secondary purposes [2]. The adaptation of EHDS is anticipated to promote access to health data for both citizens and researchers [3] and could be a step toward a data-enabled future for the European healthcare domain [4], although concerns have been raised [5–7].

The conceptualization and development of the EHDS are supported by a number of projects. From 2021 to 2023, TEHDAS, the joint action Towards the EHDS (https://tehdas.eu/tehdas1/), developed and promoted concepts for the secondary use of health data within the EHDS. In October 2022, the HealthData@EU Pilot (https://ehds2pilot.eu/) project was launched, with five use cases, to explore the potential and challenges associated with shared privacy-enabled health-data analysis infrastructures in the EU. Through these cross-border use cases, the participating institutions aimed to draw insights to inform the development and refinement of EHDS’s data-sharing framework.

This article focuses on learnings and experiences gained from one of the HealthData@EU Pilot use cases titled “Comparing Nationwide Disease Trajectories to Evaluate European Health Data Interoperability: An Application to Cardiometabolic Diseases”, later referred to as just “the use case”. The use case was a collaboration between four active academic partners across Europe (Denmark, Finland, France, and Norway). Hungary originally planned to participate, but due to a reform of the national eHealth system that led to a reorganization of responsibilities they could not participate. The overall goal of the use case was to study longitudinal health trajectories leading to cardiometabolic diseases using nationwide data from health registers and assess how these trajectories compare across the countries. In all countries, the data covered hospital encounters, medication purchases, and basic sociodemographic information, linked using pseudonymized personal identification numbers.

The use case researchers included data analysts with experience in machine learning methods applied to health outcomes and epidemiologists with experience regarding cardiometabolic diseases. All national teams had previous experience with accessing and working with centralized health registry data in their respective countries.

The overarching aim of this article is to support future projects using health data across multiple EU countries by providing a roadmap for researchers through the stages of the data user journey: discovery, access, use, and finalization.

## Methods

The teams contributing to this work played complementary roles, focusing on data analysis within the use case and on collecting relevant information for the development of the HealthData@EU infrastructure. This section outlines the steps taken from both perspectives essential to the outcomes presented.

### Data interoperability within the use case

As an initial step in the use case, a disease dictionary was developed to define disease endpoints for a set of cardiovascular diseases based on register data. The purpose was to align disease definitions across the four countries using the International Classification of Disease (10th version) (ICD-10), while taking into account country-specific coding practices. The definitions were based on earlier work with clinical expert groups [8, 9], by communicating directly with clinicians, or based on definitions used in published work [10]. Eventually, the primary outcome used in the prediction model was a composite endpoint that included diagnosis codes of ischemic heart disease and ischemic stroke. The Common Data Model Builder [11] was used to consolidate data definitions and facilitate the development of reproducible code, analyses, and model definitions prior to the data being available in each country.

### User journeys

**Figure 1.**
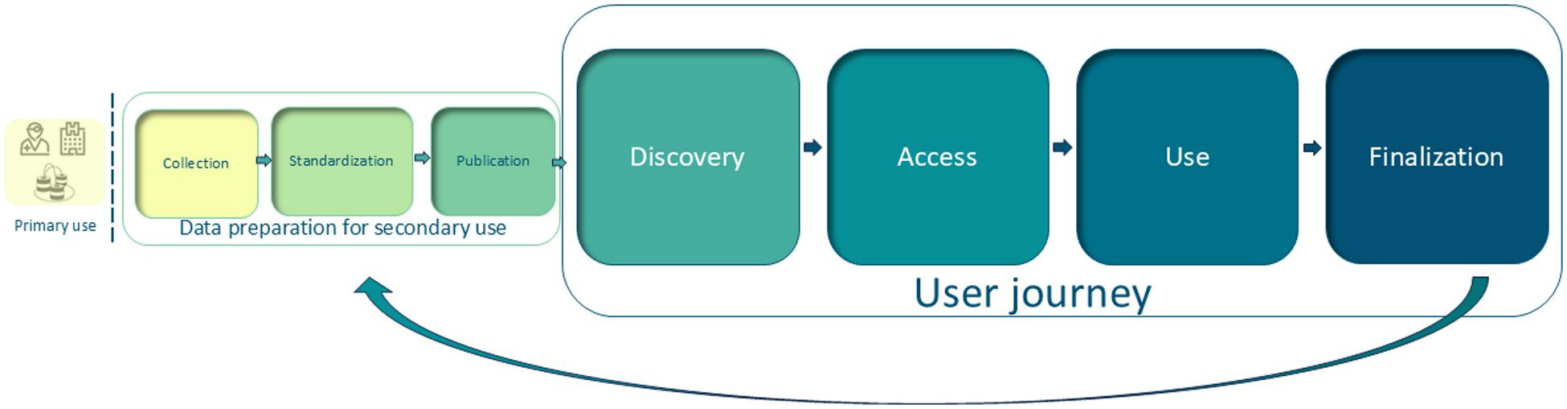
Data life cycle, highlighting the user journey, which includes discovery, access, use, and finalization.

The user journey, as conceptualized by TEHDAS, describes seven steps in the data life cycle involved in a typical situation of secondary use of health data. In this article, we focus on four of these steps involving data user perspectives: discovery, access, use, and finalization (Fig. 1) [12]. ‘Discovery’ refers to the identification of data sources a user would need to perform their work, ideally through metadata documentation that is readily accessible with searching capabilities. ‘Access’ refers to the process of gaining access to the data through appropriate regulatory means, including being granted a data permit. ‘Data use’ encompasses the activities related to the analysis of the data, and ‘finalization’ is the last phase, when the user needs to disclose the findings of their work [12]. Using the tools described below, this article provides a narrative reflection of our individual and collective experiences through the user journey.

### Learnings log and data extraction

Data were gathered from various sources, including prospectively collected points of learning documented via a structured online log. Additionally, prospective questionnaires were distributed to the analysis teams at different stages to capture relevant information and feedback. Regular meetings were held to facilitate communication and collaboration, occurring weekly among the analysis team and monthly for the entire use case. Summaries and interpretations have been provided by researchers directly involved in the project, ensuring accuracy and relevance.

### Interviews and evaluation of interviews

A core aim of the HealthData@EU Pilot project was to explore the implications and to describe the challenges encountered when analyzing cross-border data, focusing on data interoperability, quality, and security. To support this, a dedicated data work package (DWP) was established to document the actions required and identify barriers to cross-border data use. Specific actions of the DWP relevant to the use case presented here are summarized in Table 1.

**Table 1.** Actions related to the qualitative data collection

Specific action undertaken by the data work package (DWP)	Detailed explanation of action
Definition of quality measures for each use case	The group of experts within the DWP defined what quality, interoperability, and security measures should be applied in each use case throughout the data lifecycle. This included an explanation of why these measures were necessary
Collection of measures from the use cases	Participation in regular use case meetings. Involvement of some use case members in regular and additional meetings. Scheduling *ad hoc* meetings and interviews, utilizing working materials such as templates or questionnaires to gather inputs
First dedicated interview (February 2023)	Key questions were prepared by the DWP to guide the interview
Data interoperability dedicated meetings (April 2023–)	Discussions focused OMOP and PHIRI data interoperability approach, led by the IAC team
Second dedicated interview (October 2023)	Further in-depth interviews were conducted, utilizing a predefined template with specific questions to address critical issues
Feedback gathering (March 2024)	Feedback was collected for two major milestones related to data interoperability, quality, and security
Feedback gathering (May, June, July, August 2024)	Additional feedback sessions were conducted, particularly focusing on the data security milestones

DWP, data work package; IACS, Institute for Health Sciences in Aragon; OMOP, Observational Medical Outcomes Partnership; PHIRI, The Population Health Information Research Infrastructure.

Semi-structured interviews with project coordinators and analysts were a key method to gather insights. These interviews were designed to systematically capture data-related knowledge. The interviews followed a flexible guide covering topics such as data preparation, interoperability challenges, quality needs, and practical lessons learned. While structured, the interviews also allowed broader reflections, ensuring both technical insights and contextual challenges were documented to inform the future infrastructure implementation.

### Ethical statement

This article includes non-identifiable, project-level information shared by team members regarding procedures used in their research. The information presented reflects operational practices discussed in a professional context, without involving the collection of personal data or identifiable input, therefore formal ethical approval was not sought.

### Data availability

The logs and summaries of interviews that were used in this article are published with the final report from the HealthData@EU project.

## Results

**Figure 2.**
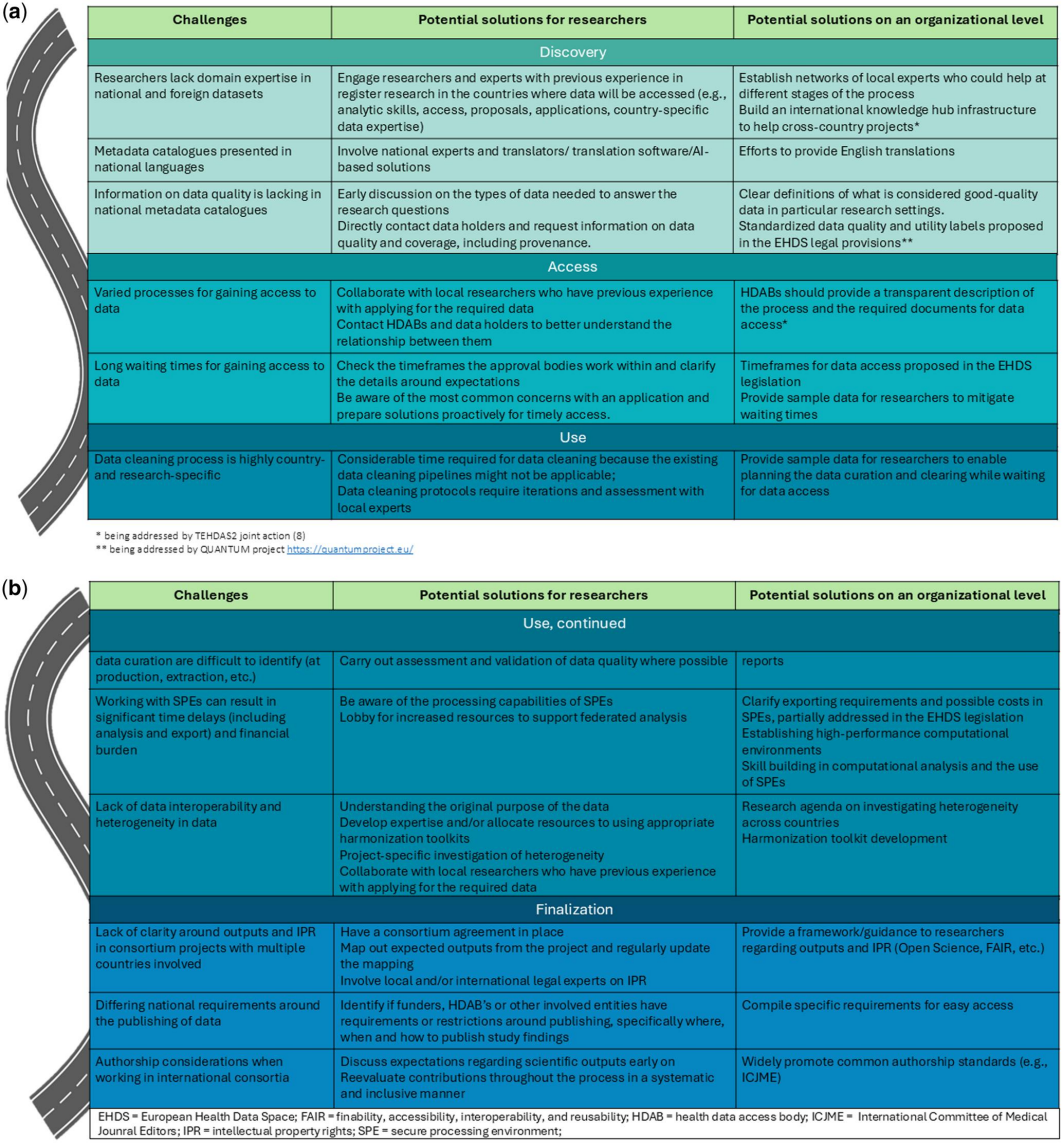
(a, b) Challenges and potential solutions for researchers along the user journey, divided into the four stages of the user journey: discovery, access, use, and finalization.

For each of the subheadings below, Fig. 2a and b highlights the major challenges and offers potential solutions at the researcher level as well as the organizational level.

### Discovery

The protocol for the use case was developed by leveraging ongoing research and existing protocols in two of the participating countries, which already had an understanding of the available data required to address the research questions. The participation of other countries was facilitated through meetings and discussions to ensure they could access the required data.

The identification of data sources relied primarily on the expertise of country-level teams, supplemented by metadata catalogs (Finland: https://aineistokatalogi.fi/catalog; Norway: https://helsedata.no/en/; France: https://www.snds.gouv.fr/SNDS/Open-Data; Denmark: https://www.dst.dk/extranet/forskningvariabellister/Oversigt%20over%20registre.html). While catalogs, often in local languages with partial English translations, provided a starting point, direct engagement with data holders proved crucial. This direct contact was essential to assess data quality and coverage, aspects lacking in most of the national metadata catalogs.

To ensure comprehensive data utilization, early discussions within the research team focused on precisely defining the necessary data types. Understanding local data required not only adherence to common data standards, as envisioned by EHDS, but also direct interaction with data holders and domain experts like clinicians. This engagement was vital for accurate variable interpretation, data coverage assessment, and navigating diverse local coding practices.

### Access

Researchers were familiar with the data access application process from previous experience, rather than national policies or availability of guidelines for accessing health data for secondary use.

The process and legal basis for gaining access to data varied between the different countries. Additionally, interpretation of the General Data Protection Regulation (GDPR) varied between countries in terms of data access. For example, data minimization was a main factor in approving a data permit in Norway and France. In Norway, the focus was on granularity of the variables, while in France, it focused on decreasing the sample size. It is essential that those applying for the data only request variables they will be using, have thoroughly considered how they will be using the data, and if they are requesting the appropriate level of granularity [13]. Ethics committees were not directly involved in the secondary use of health data projects in Finland and Denmark. For those that did require it, the process of applying for ethical approval could have been separate from the data access application, such as in Norway, or included within it, such as in France. In Norway, the Ministry of Health has drafted a legislative change to the Health Research law to remove the requirement of additional ethical approval by a regional ethics committee for pure registry-based research projects [14].

In Finland and Denmark, the participating universities already had established routes to access registry data. The experience of data access was therefore different from others without such protocols. In France and Norway, we applied for ethical approval and data access through the national pathways. Although these processes are established in some countries, they will be evolving in all European Member States as a response to the implementation of the EHDS regulation.

Waiting times for approval of the data permit and data delivery varied widely between countries, mostly due to the differences in the application process (institutional access vs. centralized application process). Most of the countries had written guidance on how long to expect applications to be handled. Researchers should be aware of potential caveats and deviations to the expected timelines, and the fact that these timelines are often regarding first addressing an application and do not include additional times researchers may need to address or change an application, or the delivery of the data after the data permit is granted.

As of the beginning of this project in 2022, researchers could expect about 6 months to get approval for the data permit and then another 6 months to get the data delivered, although this can vary widely. Under EHDS regulation, there will be certain time expectations for data access, but we expect there to still be variations in the process due to local resources.

In Norway, we experienced barriers to access when it came to data minimization and data protection issues. Alterations to the Norwegian data access application had to be implemented regarding the granularity of diagnosis codes and the use of exact dates that could risk the re-identification of patients. In France, there was no precedent in accessing the full population’s data, as originally requested, and the secure processing environment (SPE) was deemed unsuitable due to foreign ownership increasing the risk of data breaches. Therefore, the application was amended to include a random sample of 12 million people from the population. These issues can be identified early in project development through close contact with researchers with local knowledge of the data and culture around the interpretation of data security regulations.

### Use

A key element of maintaining identical data analysis processes across countries was by developing and using a common data model (CDM), tailored to our specific purpose. The CDM was developed by building on the data formats and structures from two countries that already had access to the data. In addition, a minimum data set was defined based on the data availability in all countries. Since all countries involved used the ICD-10 classification system and the Anatomical Therapeutic Chemical classification system (ATC) for prescribed medications, variable mapping was not needed. Due to time and budget constraints, any widely adopted CDM, such as the Observational Medical Outcomes Partnership (OMOP) CDM, was not used. Also, OMOP is only appropriate in some research contexts and is mainly valuable in connection with the Observational Health Data Sciences and Informatics tools, and therefore may not reflect an economical solution to adopt at the organizational level for national health data.

Building on the CDM and by utilizing Common Data Model Builder [11], the use case generated mock data based on a pre-specified common format. The mock data did not otherwise preserve information of the real data and could not confer useful findings on its own. The mock datasets were particularly valuable for defining and testing our CDM, as well as for developing an analysis pipeline. Some countries gained access to the data after 18 months, leaving less than 6 months to complete the analyses. The common data format was instrumental in preparing analysis-ready data once access was granted.

In all countries, data were delivered for use in SPEs. It is important to have *a priori* understanding of the different features of SPEs in terms of access, functionality, regulations, and costs. One of the main challenges during data preparation was computational constraints, especially when training machine learning algorithms. Extra computational resources were requested, and the process of obtaining it was lengthy and unclear. To mitigate these issues, analysts worked with subsets of the data and developed the analysis based on mock data compliant with the predefined model.

Quality controls and data cleaning were performed internally within each country and also with external validation by comparing distributions and estimates across countries. In two countries, problems found during quality control led to an additional need for data extraction and preparation. Data cleaning was highly country-specific. This made it impossible to leverage cleaning procedures from other countries or plan ahead prior to data access. Different terminology used across countries also posed challenges for interoperability, as did varying coding practices. The interpretation of these differences required deeper domain expertise to differentiate from errors.

Analysis collaboration required regular data exports and the practicalities varied across countries. No individual-level data could be exported from the SPE, but exporting aggregated data was allowed in all countries. The minimum level of aggregation required varied according to the institutional policies between five and 10 individuals. In most countries, an external person (e.g. a person employed by an HDAB or a data controller or personnel at the SPE), independent from the researchers, was responsible for reviewing export requests and exporting the data and results. This process required time and caused significant delays. In addition, in Denmark, data exports were subject to additional costs. In Norway, more control was given to the researcher in terms of exporting and importing data to the SPE.

### Finalization

In this use case, because data were not shared across borders, specific data ownership remained with the national research groups. Early discussions focused on the ownership of outputs, such as the CDM and analysis products. It was agreed that to support knowledge sharing, all outputs could be made publicly shared as an open repository following Findable, Accessible, Interoperable, and Reusable (FAIR) principles [15]. Considerations were also given to whether HDABs or other groups would need to approve publication submissions.

Early planning emphasized the importance of discussing the article writing process and preparing a timeline to ensure timely and coordinated publication efforts. Documenting local knowledge and publicly disseminating findings are crucial for informing future projects.

## Discussion

The proposed EHDS framework has the potential to bring forth a significant step toward creating a digital health ecosystem that facilitates cross-border data sharing and public health research. Here, we highlight practical challenges related to the secondary use of data across multiple EU countries and propose solutions for researchers and organizations to address these (Fig. 2a and b). While several roadmaps have been developed in recent years to streamline research processes [16–19], the goal of this article was to create a targeted roadmap specifically for researchers undertaking cross-European projects with registry data.

As others also noted, our observations revealed that technical and logistical issues, particularly those related to data access, interoperability, and quality, present a significant obstacle to EHDS's successful implementation [4, 20, 21]. Given differences in how medicine is practiced, including the underlying incentives, and how medical practices are further coded in various national systems, harmonizing health data across borders remains a challenge. For example, while standards such as ICD-10 are widely used, the coding practices vary by country, even when based on the same diagnostic guidelines, potentially leading to different reflections of population health [22].

To address this challenge, we propose a systematic research agenda focused on identifying variances and biases in European datasets and clinical practice, supported by sufficient resources that reflect its importance. A crucial aspect of this agenda would involve domain experts determining whether observed data differences are indicative of real differences in health or healthcare processes, or the result of technical inconsistencies and data artifacts such as coding practices with the purposes of reimbursement versus quality-of-care monitoring.

Data provenance, which involves tracing the origin and journey of health data, is essential for ensuring accurate interpretations of clinical data across countries. Provenance information helps clarify how data was collected, whether schemas have changed, and any limitations associated with the data, ensuring that researchers understand the context behind the datasets. Involving experts who are familiar with country-specific registries and coding conventions can assist in clarifying data nuances. Such experts may not always be available, and EHDS has even removed national provisions demanding local expertise. The hope is that standardized documentation and metadata that reflects the quality, integrity, and reliability of the datasets, as developed in QUANTUM [23], can do part of this work. However, our use case points to the limits of relying on metadata and highlights the requirement for local expertise. We therefore emphasize the need for a research agenda exploring variance in documentation practices which can enhance the interpretative ability of the health data research community.

Overall, we find it critical that knowledge on European health data is collected and curated on a large scale, and we would see it beneficial if EHDS knowledge hubs similar to successful infrastructures like the European Life Sciences Infrastructure (ELIXIR) and BBMRI-ERIC were established [24, 25]. As HDABs will have the obligation to educate on the use of health data, these bodies could be at the forefront of the establishment of such infrastructures. We cannot understate the importance of enlisting local expertise including clinicians’, data holders’, and data analysts’ perspectives to properly understand data. Without soliciting such collaborations, researchers will risk missing important data contexts (e.g. related to country-specific legislation, coding conventions, and temporal changes). Furthermore, it is critical to develop and share open-source pipelines and tools to ensure the reproducibility of analyses across countries following FAIR principles [15].

The EHDS must account for significant cultural, institutional, and infrastructural differences, as well as technical readiness, between the participating countries. For example, the Nordic countries have been cited as a model for health data governance due to their extensive use of national registries and collaborative efforts in precision medicine initiatives [26, 27]. The success of such a framework will largely depend on each country's healthcare infrastructure, workforce information, and communication technology competence, and capacity to address cultural differences. At earlier stages, countries with a longer tradition of secondary use of health data are likely to be more active under EHDS [28], while others may lag behind, risking the amplification of regional inequalities in health data usage. In this respect, 30 European countries are involved in TEHDAS2 (https://tehdas.eu/), developing guidelines and technical specifications for harmonized EHDS implementation.

This article is largely based on the experiences in Nordic countries, which have a long history of secondary use of health data, with established processes for accessing and using register data. In France, similar processes have been put in place in recent years under the direction of Health Data Hub. Therefore, the experiences and challenges we encountered reflect issues arising in the presence of streamlined practices. This use case focused on health registry data-based epidemiology, and may not be fully applicable to other areas of health research, but we believe our findings related to the importance of understanding and appreciating variance between countries remains valid.

Through the experience of this project, the use case team recognizes that a two-year timeframe is likely insufficient for registry-based studies at the current set-up. The EHDS regulation aims to shorten such timeframes for access to health data, but there are many factors that need to be optimized for this to work. Regulation standards need to be considered for issues of intellectual property rights, the reusability of common resources, including secured funding, and the dissemination of knowledge. These key elements need careful management to maximize the impact and sustainability of research outputs.

We acknowledge that one of the primary aims during the early years of EHDS is data digitization, and therefore some of our recommendations might be more topical in later years. Yet, our perspective is diverse, and each country encountered a set of problems despite their practices being built on their own experiences. We believe that addressing these challenges in the early phase with regulated solutions will ensure high-quality research and save considerable effort for the future.

Conflict of interest: A.G. is the founder of Real World Genetics Oy. C.H. is a senior adviser at the decision authority on access to Norwegian health data for secondary use, the Health Data Service. This authority is likely to be appointed the Norwegian coordinating HDAB following the implementation of the EHDS. All other authors report no conflict of interest.

## Funding

The resources in the use case are funded by the European Health and Digital Executive Agency (HADEA, grant number: 101079839) (“EU executive agency” or “granting authority”), under the powers delegated by the European Commission (“European Commission”), and its relevant national partners in the EHDS2 pilot. Denmark: SUNDHEDSDATASTYRELSEN (DHDA), PIC 888310675, established in ORESTADS BOULEVARD 5, COPENHAGEN 2300, Denmark. (GRANT NR). Finland: TERVEYDEN JA HYVINVOINNIN LAITOS (Findata/THL), PIC 996697893, established in MANNERHEIMINTIE 166, HELSINKI 00271, Finland. France: PLATEFORME DES DONNEES DE SANTE (Health Data Hub), PIC 895824586, established in 9 RUE GEORGES PITARD, Paris 75015, France. Norway: HELSEDIREKTORATET (NDH), PIC 974772304, established in VITAMINVEIEN 4, OSLO 0213, Norway. The Copenhagen Health Complexity Center (A.G.Z., N.H.R., T.V.V.) was funded by TrygFonden. T.V.V. was supported by the “Data Science Investigator—Emerging 2022” grant from the Novo Nordisk Foundation (NNF22OC0075284). R.B.F. and C.H. were supported by the European Health and Digital Executive Agency (HADEA) EU4H action grant for EHDS2 Pilot (101079839) as well as by additional funding from the Norwegian Institute of Public Health and the Norwegian Directorate of Health. A.L.V. was supported by the HADEA action grant for EHDS2 Pilot (101079839) and the grant from the Research Council of Finland (336357). K.H. was funded by the European Union. This work was supported by ERC grant DataSpace, grant agreement number 101096999.

## Disclaimer

Views and opinions expressed are, however, those of the author(s) only and do not necessarily reflect those of the European Union or the European Health and Digital Executive Agency (HADEA). Neither the European Union nor the granting authority can be held responsible for them.

Key points

- The European Health Data Space (EHDS) has the potential to transform cross-border health research in Europe, but technical and regulatory challenges, particularly around data interoperability and access, remain barriers.
- Variations in national data coding systems, such as differing applications of ICD-10, complicate the harmonization of health data across European countries, affecting research quality and consistency.
- Close collaboration with local experts will be important in ensuring accurate interpretation of national health datasets, and data variability needs to become acknowledged as a research area in need of development.
- Establishing centralized knowledge hubs and improving data provenance standards are important for supporting large scale, cross-country research projects within the EHDS.
- Public health policy should prioritize streamlining data access procedures and enhancing computational resources in secure environments to enable more timely and effective use of health data across Europe.
